# Levels of Circulating Ketone Bodies in Patients Undergoing Cardiac Surgery on Cardiopulmonary Bypass

**DOI:** 10.3390/cells13040294

**Published:** 2024-02-06

**Authors:** Anja Levis, Markus Huber, Déborah Mathis, Mark G. Filipovic, Andrea Stieger, Lorenz Räber, Frank Stueber, Markus M. Luedi

**Affiliations:** 1 Department of Anaesthesiology and Pain Medicine, Inselspital, Bern University Hospital, University of Bern, 3010 Bern, Switzerland; markus.huber@insel.ch (M.H.); mark.filipovic@insel.ch (M.G.F.); frank.stueber@insel.ch (F.S.); markus.luedi@extern.insel.ch (M.M.L.); 2 University Institute of Clinical Chemistry, Inselspital, Bern University Hospital, University of Bern, 3010 Bern, Switzerland; deborah.mathis@insel.ch; 3 Department of Anaesthesiology, Pain- and Rescue-Medicine, Cantonal Hospital of St. Gallen, 9007 St. Gallen, Switzerland; andrea.stieger@kssg.ch; 4 Department of Cardiology, Bern University Hospital, University of Bern, 3010 Bern, Switzerland; lorenz.raeber@insel.ch

**Keywords:** ketone bodies, acetoacetate, 3-hydroxybutyrate, ischemia/reperfusion, cardiopulmonary bypass

## Abstract

Ketone bodies (KBs) are energy-efficient substrates utilized by the heart depending on its metabolic demand and substrate availability. Levels of circulating KBs have been shown to be elevated in acute and chronic cardiovascular disease and are associated with severity of disease in patients with heart failure and functional outcome after myocardial infarction. To investigate whether this pattern similarly applies to patients undergoing cardiac surgery involving cardiopulmonary bypass (CPB), we analysed prospectively collected pre- and postoperative blood samples from 192 cardiac surgery patients and compared levels and perioperative changes in total KBs with Troponin T as a marker of myocardial cell injury. We explored the association of patient characteristics and comorbidities for each of the two biomarkers separately and comparatively. Median levels of KBs decreased significantly over the perioperative period and inversely correlated with changes observed for Troponin T. Associations of patient characteristics with ketone body perioperative course showed notable differences compared to Troponin T, possibly highlighting factors acting as a “driver” for the change in the respective biomarker. We found an inverse correlation between perioperative change in ketone body levels and changes in troponin, indicating a marked decrease in ketone body concentrations in patients exhibiting greater myocardial cell injury. Further investigations aimed at better understanding the role of KBs on perioperative changes are warranted.

## 1. Introduction

Ketone bodies are an alternative energy source for cardiac metabolism and, together with amino acids and lactate, are responsible for 5–20% of adenosine triphosphate (ATP) production in the healthy heart [1,2]. There is a substantial body of evidence suggesting that in the context of heart failure, elevated plasma concentration and consequently increased systemic availability of circulating KBs lead to increased myocardial uptake and a metabolic shift towards ketone body oxidation as an energy-efficient source for ATP production [3]. The evidence indicates that this mechanism is an adaptive part of the stress response [4]. Moreover, the severity of heart failure appears to correlate with elevated circulating ketone body concentrations [5].

Based on these findings, there is growing interest in KBs as possible diagnostic and prognostic markers as well as in their potential therapeutic role [6,7]. The role of KBs in the setting of ischemia/reperfusion is not established. In line with the well-documented changes occurring in heart failure, recent studies found elevated levels of circulating KBs in patients with acute ST-elevation myocardial infarction [8] and in mice following left anterior descending artery ligation surgery [9]. In both studies, elevated KBs were negatively associated with left ventricular function [8,9].

However, little is known about the contribution of KBs to cardiac metabolism in the patient undergoing cardiac surgery or to possible changes in the setting of cardiopulmonary bypass (CPB) and cardioplegia.

The aim of this report was to investigate the longitudinal changes of KBs in patients undergoing cardiac surgery including CPB and to compare levels and perioperative changes with a well-established biomarker for cardiac cell damage, Troponin T. We hypothesized that levels of circulating KBs would increase following CPB surgery in a manner similar to patients with acute myocardial infarction and reperfusion and would correlate with markers of myocardial cell injury. We assumed a positive association for the perioperative increase with aortic cross clamp duration as a surrogate for ischemic burden during cardioplegic arrest. The primary readout was the change in total concentration of two main KBs (3-OH-hydroxybutyrate and acetoacetate) in peripheral blood from preoperative baseline to 24 h postoperatively. We observed a significant decrease in total ketone body levels assessed preoperatively and 24 h postoperatively, which negatively correlated with changes in troponin and were associated with greater myocardial cell injury.

## 2. Materials and Methods

### 2.1. Study Population

This retrospective analysis included 192 patients within the Bern Perioperative Biobank (ClinicalTrials.gov; NCT04767685), a previously described prospective observational cohort study in adult patients undergoing elective cardiac surgery [10,11]. The number of patients analysed for this study was defined by the preexisting database and consisted of a non-probabilistic sampling of consecutive patients meeting the inclusion criteria. The study was approved by the local ethics committee (Cantonal Ethics Commission of Bern, Bern, CH-KEK Nr. 2018-01272 for sampling and KEK Nr. 2019-2000 for data analysis), and informed consent was provided prior to study inclusion. Inclusion criteria were adult patients with consent undergoing elective cardiac surgery including coronary artery bypass grafting (CABG), isolated or combined with replacement or repair of the aortic (AVR), mitral (MVR), and tricuspid valves, as well as surgery of the ascending aorta and aortic arch. Emergency surgery, children, women with suspected or confirmed pregnancy, and patients without consent were excluded. Surgical access through sternotomy and CPB was performed in all patients, either with conventional extracorporeal circulation circuits (CECCs) or minimally invasive extracorporeal circulation circuits (MIECCs) [12].

### 2.2. Blood Sampling and Analysis

Blood samples (EDTA) collected before induction of general anaesthesia (preoperative) and 24 h after surgery (postoperative) were immediately transported to the Bern Liquid Biobank and frozen at −80 °C within one-half hour from sampling. Routinely used cardiac biomarkers were analysed at the University Institute of Clinical Chemistry, Inselspital, Bern University Hospital according to standardized routine laboratory methods. The KBs 3-hydroxybutyrate and acetoacetate were analysed using liquid chromatography mass spectrometry (LC-MS/MS, Shimadzu Nexera coupled to AB Sciex 6500+ Qtrap) with adaptations of a published protocol. In short, 50 μL of plasma was precipitated with 50 μL of 0.6 M perchloric acid solution containing the internal standards. Samples were vortexed, kept on ice for 15 min, centrifuged, and supernatants transferred to HPLC-vials. A total of 2 μL was injected into the LC-MS system and KBs were analysed in negative ion mode as described previously. The method working range (calibration range) was of 10–3000 µmol/L and lower limit of quantification (LLOQ) of 5 µmol/L for both KBs. Inter-day precision (coefficient of variation, CV) at 20 µmol/L was of <15% and <20% for 3-hydroxybutyrate and acetoacetate, respectively. At 100 µmol/L and higher concentrations, precision was of <15% for both KBs. The method accuracy was checked with external quality controls for 3-hydroxybutyrate and with inter-laboratory comparison for acetoacetate.

### 2.3. Data collection

Relevant pre-, peri-, and postoperative data were collected from electronic patient charts (Dendrite Clinical Systems Ltd., Henley on-Thames, UK). Information on all-cause mortality was sourced from internal hospital records or national records. Presumed risk of 30-day all-cause mortality was assessed by calculating EuroSCORE II ^2^.

### 2.4. Study Outcomes

The primary outcome was preoperative level and perioperative change of circulating total KBs. The primary endpoint was association of KB levels and trajectory with hs-Troponin. Exploratory analyses included investigation of associations of patient characteristics and comorbidities with total KB and troponin, for both preoperative values and for perioperative changes.

### 2.5. Statistical Analysis

Categorical variables were summarized with counts and frequencies. Continuous variables were summarized with mean and standard deviation in cases of normally distributed variables and with median and interquartile range (IQR) otherwise. Normality of numerical variables was assessed with the Shapiro–Wilk test. Group comparisons with respect to low and high preoperative total ketone bodies based on the median value were computed using chi-square or Fisher’s exact test for categorical variables and by Student’s *t*-test or unpaired two-sample Wilcoxon test for numerical variables.

Data availability is presented in the Tables.

Perioperative changes of the biomarkers were computed with the paired-sample Wilcoxon test to account for the repeated measures design of this study; the median change and associated 95% confidence intervals based on R’s Wilcoxon test function are shown.

The associations of patient demographics and comorbidities with both total ketone bodies and troponin measured pre- and postoperatively were assessed with a multivariable regression with standardized (mean and standard deviation) covariates and dependent variables: the standardization allows for removal of the different units of the variables and comparison of the associations (e.g., the regression coefficients) on the same scale. To account for biomarker levels covering multiple orders of magnitude, log-transformed preoperative levels were used in the multivariable logistic regression. The associations between aortic cross-clamping and perioperative changes in ketone bodies and log-transformed troponin were assessed with linear regression.

A *p* < 0.05 was considered statistically significant. Given the exploratory nature of our study, no *p*-value adjustment for multiple comparisons was performed. All computations were performed with R version 4.0.2 [13].

## 3. Results

Patient and surgical characteristics and longitudinal changes of KBs and hs-Troponin T are provided in Table 1 and Table 2.

A total of 192 patients were included in the study (Table 1). Median age was 67 years (interquartile range 60, 73), median body mass index (BMI) was 26.1 kg/m^2^ (IQR: 23.7, 30.4), and a majority of patients were male (75.5%). Arterial hypertension was present in 68.4% of patients, dyslipidaemia in 58.1%, and insulin-dependent diabetes in 18.2%. The most common procedures included AVR (44.8%) and CABG (40.1%), followed by MVR (23.4%) and ascending aortic replacement (19.8%). In 22.4% of cases, patients were on MiECC. Median aortic cross clamp duration was 68.5 min (IQR: 52.0, 91.8).

**Figure 1:**
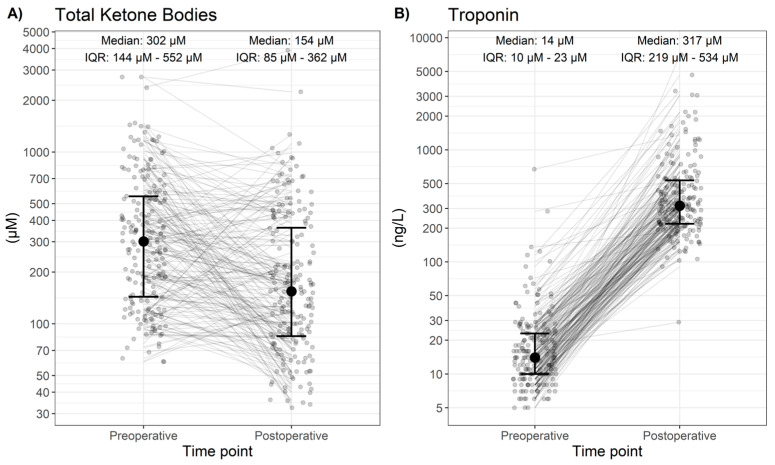
Pre- and postoperative measurements of total KBs and troponin. Individual patients are shown as coloured dots. Summary measures (median and interquartile range) for each time point are shown in Table 2. Pre-and postoperative measures are shown for (**A**) total ketone bodies; **(B)** troponin.

The median of total ketone bodies showed a significant decrease over the perioperative course (−123 (−175 to −79) µM, *p* < 0.001, Table 2, Figure 1A). In contrast, median hs-Troponin-T showed a significant increase (+340 (302 to 391) ng/L, *p* < 0.001, Table 2, Figure 1B).

**Figure 2:**
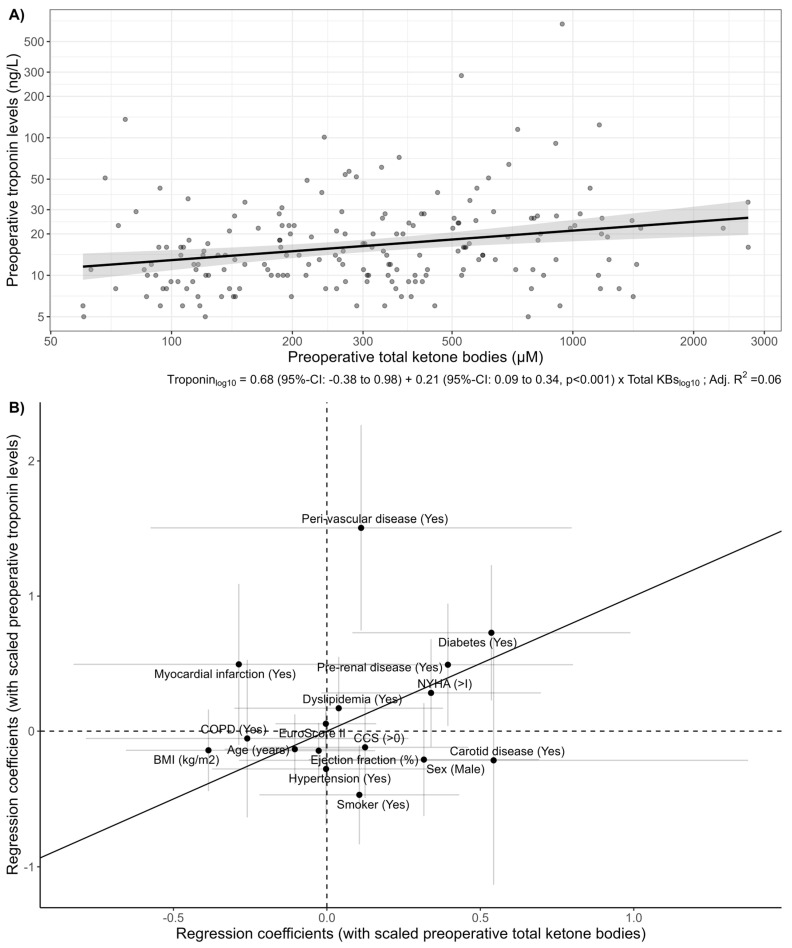
(**A**) Scatterplot of preoperative total ketone bodies and troponin levels. Each dot represents a patient. A linear regression fit with the mean estimate (solid line) and 95% confidence interval (shaded) are shown. Regression coefficients are shown below the *x*-axis. (**B**) Scatterplot of regression coefficients (denoting adjusted associations) of scaled patient characteristics and comorbidities with preoperative total KBs and troponin (see Methods). The solid diagonal line refers to a 1:1 relationship, with the distance from this line serving as a visual indicator of variations in the strength of associations between patient characteristics and preoperative levels of each of the biomarkers.

Figure 2A highlights a positive, linear association of preoperative total KBs with troponin (*p* < 0.001). The adjusted associations of scaled patient demographics and comorbidities with preoperative total KBs and troponin levels were similar (Figure 2B). However, there were notable differences. For example, the association of sex with preoperative total KBs was stronger than the association with preoperative troponin levels.

**Figure 3:**
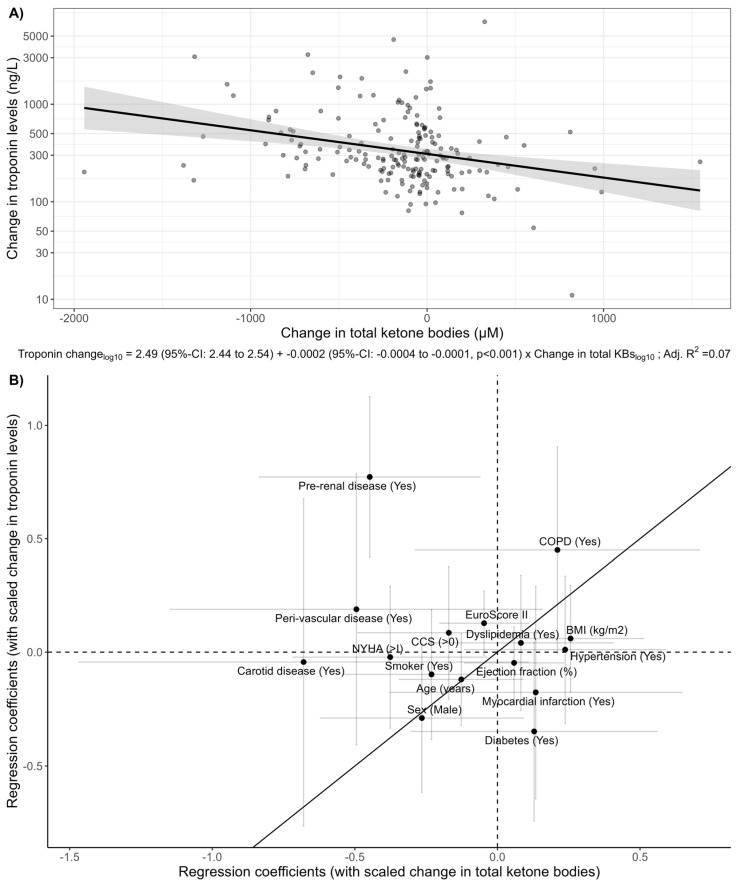
(**A**) Scatterplot of perioperative changes in total ketone bodies and troponin levels. Each dot represents a patient. A linear regression fit with the mean estimate (solid line) and 95% confidence interval (shaded) are shown. Regression coefficients are shown below the *x*-axis. (**B**) Scatterplot of regression coefficients (denoting adjusted associations) of scaled patient characteristics and comorbidities with perioperative change in total KBs and troponin (see Methods). The solid diagonal line refers to a 1:1 relationship, with the distance from this line serving as a visual indicator of variations in the strength of associations between patient characteristics and perioperative changes for each of the biomarkers.

Figure 3A highlights a negative, linear association of the perioperative change in total KBs with the perioperative troponin changes (*p* < 0.001). The adjusted associations of scaled patient demographics and comorbidities with perioperative changes in total KBs and perioperative troponin levels show large differences (Figure 3B), possibly highlighting different physiological drivers of the change in total KBs and troponin levels.

**Figure 4:**
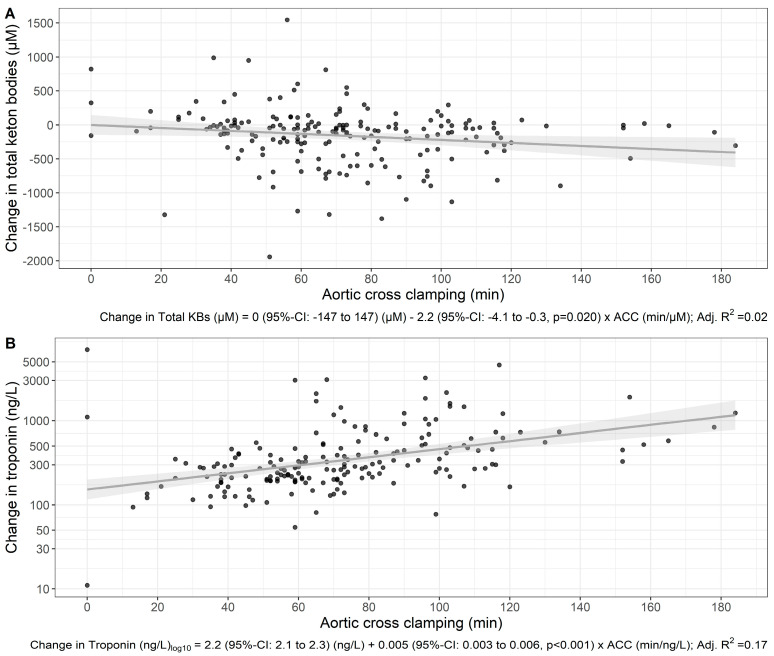
Scatterplot and linear regression of perioperative changes in total ketone bodies (**A**) and in troponin levels (**B**). Each dot represents a patient. A linear regression fit with the mean estimate (solid line) and 95% confidence interval (shaded) are shown. The regression coefficients are shown below the *x*-axis.

After adjustment for cardiotomy, increased troponin correlated with aortic cross clamp duration (Figure 4, *p* = 0.023, Supplementary Figures S1 and S2, Tables S1 and S2). After adjustment for aortic cross clamp duration, the perioperative increase in troponin was not different for patients undergoing a procedure involving cardiotomy compared to those without (*p* = 0.12, Supplementary Figure S2 and Table S2). In contrast, a change in median levels of total ketone bodies was not associated with duration of aortic cross-clamping (*p* = 0.056, Supplementary Tables S1 and S2) and did not differ for patients with or without cardiotomy (Figure 4, *p* = 0.5, Supplementary Table S2).

The grey bars denote the uncertainty range, suggesting a notable influence for the respective factor in determining preoperative levels of either troponin or ketone bodies if they do not intersect the corresponding dotted “0” line.

The grey bars denote the uncertainty range, suggesting a notable influence for perioperative change in concentration of the respective factor if they do not intersect the corresponding dotted “0” line.

## 4. Discussion

In the current analysis we observed a significant decrease in total ketone body levels assessed preoperatively and 24 h postoperatively. In addition, we found a negative linear correlation of perioperative changes in ketone body levels with changes in troponin, indicative of a more marked decrease in ketone body concentrations in patients exhibiting greater myocardial cell injury.

These findings contrast with those in the cardiologic literature. In general, plasma levels of circulating KBs are elevated in physiological conditions characterized by limited availability of carbohydrates such as prolonged fasting and exercise [14], but also in situations of oxidative stress, as a response to catecholamine release, and in numerous pathologic conditions such as poorly controlled type 1 diabetes [15]. In heart failure, elevation of circulating KB concentrations is associated with severity of disease [5]. Similarly, elevation of KB concentration in the setting of acute ST-elevation myocardial infarction has been shown [8]. Interestingly, the increase observed 24 h after reperfusion was associated with larger infarct size and was inversely proportional to LV function at the 4-month follow up. Alongside increased availability in the circulation, enhanced cardiac uptake and utilization of KBs has been suggested to be a common and adaptive cardiac response to stress [4,16].

Factors specific to the perioperative setting may contribute to divergent observations in the current analysis. While our study primarily concentrated on examining ischemia and reperfusion, it is important to note that cardioplegic arrest during extracorporeal circulation presents variations from the ischemia/reperfusion scenario observed in the context of myocardial infarction with reperfusion therapy. It is conceivable that pathophysiological reactions to the ischemic burden are not directly comparable. In addition, even for comparable duration of cardioplegic arrest, the extent of ischemia/reperfusion injury may vary, based on individual features of the underlying cardiac condition, influencing susceptibility to ischemic stress. Surgical and technical aspects such as the effectiveness of cardioprotection or partially clamping of the aorta while proximal anastomoses are completed further contribute to the heterogeneity of surgical characteristics.

Nevertheless, in our study we confirmed the association of the duration of aortic cross-clamping and the perioperative change in troponin, highlighting the role of ischemia/reperfusion on the observed increase in this marker of myocardial cell injury. This has previously been shown in a larger dataset with 1200 patients undergoing first time myocardial revascularization, where the relative risk of postoperative myocardial infarction (judged by serial electrocardiograms and release of enzymes) increased continuously with the duration of aortic cross-clamping [17,18]. In contrast, the absence of a discernible association with the perioperative change in ketone body levels indicates that the duration of aortic cross-clamping has a relatively diminished impact on ketone bodies, pointing towards distinct physiological drivers for these two markers.

Multiple factors other than ischemic burden during cardioplegic arrest contribute to the cumulative amount of myocardial cell injury measured postoperatively. In addition to the possible variations in vulnerability to ischemia described above, the amount and type of stress additionally encountered depends on underlying cardiac pathology and type of surgery. For example, cardiac workload is substantially reduced when an obstructed aortic valve is replaced, whereas a preoperatively seemingly “normal” ventricle may suddenly appear reduced in function once faced with the increased afterload after correction of a regurgitate atrioventricular valve or after fail and dilate, thus adding further wall stress to the heart recovering from cardioplegia. Specific to the cardiac surgery patient, surgical trauma inflicts direct myocardial damage, but with different procedures the extent of direct tissue damage varies, e.g., isolated coronary artery revascularization vs. cardiotomy in a hypertrophic heart. The extent to which each of these factors contributes to the resulting myocardial injury, and how this reflects in the levels of circulating total ketone bodies in this heterogeneous study cohort encompassing various types of surgery, is not known.

Against our expectations, a perioperative increase in troponin was not significantly enhanced in “open heart” surgery (i.e., procedures including cardiotomy), despite the additional direct surgical damage, compared to procedures limited to structures situated and accessed superficial to the heart muscle (e.g., isolated coronary artery bypass grafting). Similarly, cardiotomy performed as part of the surgical procedure did not influence perioperative changes in ketone body levels. Cardiotomy seems not to be an important variable for course of either Troponin T or ketone bodies.

Notably, while we found a decrease in median ketone body levels, the direction of change was inconsistent. The limited number of patients precluded stratification based on divergent trajectories of ketone body levels and subsequent analyses regarding potential denominators for the direction of change.

While limited sample size precluded robust regression analysis, computing adjusted associations enabled the formation of an impression regarding the cumulative impact of patient characteristics on the observed outcomes. Variations in the strength of these associations between the two biomarkers provide a basis for generating additional hypotheses about specific physiological drivers. As depicted in Figure 2B, preoperative values closely align along the diagonal line of identity, suggesting a consistent impact of patient characteristics and comorbidities on preoperative ketone body and troponin levels. However, Figure 3B highlights significant differences in adjusted associations during the perioperative course. This implies that the factors under investigation may not have uniform effects on the trajectories of these two biomarkers and distinct factors within the evaluated variables contribute to these changes. For instance, diabetes influences preoperative ketone body levels, while smoking does not (Figure 2B). Pre-existing renal insufficiency plays a significant role in the change of ketone bodies, whereas diabetes does not have a similar impact (Figure 3B).

Our study is subject to limitations inherent to retrospective and observational design. The preoperative concentrations of the ketone bodies may not reflect a baseline comparable to reported levels in other studies, where baselines were obtained in the outpatient setting and fasting status was not reported. Since an increase in ketone bodies is a physiologic response to fasting, and only scheduled cases were included in this study, variable periods of preoperative fasting are likely to have influenced KB levels obtained immediately before induction of anaesthesia. Similarly, the 24 h samples were drawn at different timepoints relative to extubation, so oral intake as well as amount and type of fluid administered prior to the 24 h blood sampling may vary. The heterogeneity of the procedures precluded standardized postoperative fluid management in all patients, so fluid balances in this patient population are likely inconsistent and may exert effects on the actual concentration as well as interference with precision of the laboratory assay of ketone bodies. Based on the data from this retrospective analysis, it is impossible to determine the extent to which each individual blood sample was affected by these factors.

The same is true for a variety of factors that could not be controlled in this analysis, as the blood KB concentration underlies multiple factors such as circadian rhythm, nutritional and hormonal status, form of diet, and intake of certain medications [19,20]. Diabetes is a common comorbidity and was represented in 18.2% of our study population, and almost half of the patients (44.8%) were preoperatively treated with betablockers, a common part of medical management in patients suffering from cardiovascular disease. Both agents are known to influence circulating ketone body levels. In the descriptive analysis, we found an equal distribution of patients with and without insulin-dependent diabetes and betablockers between the high versus low ketone body group. The limited sample size precluded detailed analysis of subgroups.

Specific to cardiac surgery, the continuous administration of at least one catecholaminergic drug is common. The ketogenic effect of catecholamines in healthy and diabetic patients [21,22], as well as dependency of ketogenesis and KB metabolism on oxidative stress and substrate availability are well described. However, the extent of their influences in the very specific perioperative setting including activation of inflammatory system through CPB, variable amount and type of fluids and cardioplegia (usually containing glucose) administered, and infusion of catecholamines remains unknown.

Apart from these unaccounted confounders, absorption and filtration in the extracorporeal circuit is possible, as well as a true decrease secondary to alteration of synthesis in the liver induced by CPB. Studies assessing the ratio of acetoacetate to 3-hydroxybutyrate (arterial ketone body ratio (AKBR), reflecting hepatic energy reserve) have consistently shown a decrease in the AKBR upon initiation of CPB [23,24]. The extent of decrease and time to recovery has been shown to differ between normo- and hypothermic bypass, pulsatile versus laminar flow, and blood pressure [25], with the AKRB remaining below baseline levels until the first or second postoperative day [26]. Absolute values and trajectory of individual levels of each ketone body are not reported in most of these studies, but the factors described as influencing AKBR were present in our study population to varying degrees and cannot be controlled for in this retrospective analysis.

Furthermore, being a single-centre study, the heterogeneous population of cardiac surgical patients in our centre with a wide spectrum of procedures performed in a limited number of patients may not be comparable to the cardiological patient or cardiac surgery patients in other centres. Further, different non-standardized laboratory assays being used to measure KBs may limit comparison with other studies. However, despite all limitations, our study reflects a scenario that most closely corresponds to the real-world setting and is therefore applicable to the underlying question.

## 5. Conclusions

In summary, we found an unexpected decrease in median levels of circulating total ketone bodies over the perioperative course. This change inversely correlated with the extent of myocardial injury measured by Troponin T, a finding that contrasts with the cardiologic literature. While the study was limited by sample size, the analysis of associations pointed towards different physiological drivers for the perioperative courses of Troponin T and ketone bodies. Further studies are needed to validate these findings and to confirm the highlighted factors as potential determinants of the trajectory of ketone bodies.

## Figures and Tables

**Table 1:** Baseline patient and surgical characteristics.

Variable	All Patients	Low Preoperative Total Ketone Bodies (<Median of 302 µM)	High Preoperative Total Ketone Bodies (≥Median of 302 µM)	*p*	*n*
	*n* = 192	*n* = 96	*n* = 96		
**Age** (years)	67.0 [60.0;73.0]	65.5 [59.0;72.0]	68.0 [60.8;75.0]	0.059	192
**Sex** (female)	47 (24.5%)	21 (21.9%)	26 (27.1%)	0.502	192
**Body Mass Index** (BMI; kg/m^2^)	26.1 [23.7;30.4]	28.0 [25.0;31.7]	24.7 [22.7;28.0]	<0.001	192
**Diabetes on insulin** (Yes)	35 (18.2%)	17 (17.7%)	18 (18.8%)	>0.999	192
**Hypertension** (Yes)	130 (68.4%)	68 (71.6%)	62 (65.3%)	0.435	190
**Dyslipidaemia** (Yes)	111 (58.1%)	59 (62.1%)	52 (54.2%)	0.334	191
**Smoker**:				0.880	188
Non-smoker	97 (51.6%)	48 (50.5%)	49 (52.7%)		
Previous/current smoker	91 (48.4%)	47 (49.5%)	44 (47.3%)		
**Obesity** (Yes)	52 (27.1%)	33 (34.4%)	19 (19.8%)	0.035	192
**Preoperative renal disease** (Yes)	43 (22.4%)	13 (13.5%)	30 (31.2%)	0.006	192
**Peripheral vascular disease** (Yes)	11 (6.2%)	6 (6.7%)	5 (5.7%)	>0.999	178
**Carotid disease** (Yes)	6 (3.7%)	3 (3.9%)	3 (3.6%)	>0.999	162
**Myocardial infarction** (Yes)	20 (10.5%)	9 (9.5%)	11 (11.5%)	0.832	191
**COPD** (Yes)	23 (12.1%)	15 (15.8%)	8 (8.42%)	0.182	190
**NYHA** (>1)	131 (68.6%)	59 (62.1%)	72 (75.0%)	0.078	191
**CCS** (>0)	71 (37.6%)	33 (35.5%)	38 (39.6%)	0.666	189
**Ejection Fraction** (%)	60.0 [55.0;65.0]	60.0 [56.5;65.0]	60.0 [55.0;65.0]	0.638	191
**EuroSCORE 2**	1.73 [0.90;2.93]	1.52 [0.83;2.68]	2.19 [1.08;3.46]	0.035	184
**Betablocker (Yes)**	86 (44.8%)	43 (44.8%)	43 (44.8%)	>0.999	192
**ECC or MiECC:**				0.299	192
ECC	149 (77.6%)	71 (74.0%)	78 (81.2%)		
MiECC	43 (22.4%)	25 (26.0%)	18 (18.8%)		
**Deep hypothermic cardiac arrest** (Yes)	19 (10.0%)	8 (8.4%)	11 (11.5%)	0.646	191
**Aortic valve** (Yes)	86 (44.8%)	36 (37.5%)	50 (52.1%)	0.059	192
**Mitral valve** (Yes)	45 (23.4%)	22 (22.9%)	23 (24.0%)	>0.999	192
**Tricuspid valve** (Yes)	17 (8.85%)	7 (7.29%)	10 (10.4%)	0.611	192
**Coronary artery bypass** (Yes)	77 (40.1%)	45 (46.9%)	32 (33.3%)	0.077	192
**Ascending Aortic** (Yes)	38 (19.8%)	19 (19.8%)	19 (19.8%)	>0.999	192
**Aortic Arch** (Yes)	11 (5.7%)	5 (5.2%)	6 (6.3%)	>0.999	192
**Bypass time** (min)	104 [80.0;132]	100 [82.8;133]	108 [80.0;132]	0.793	192
**Aortic cross-clamping** (min)	68.5 [52.0;91.8]	68.0 [49.8;95.5]	69.0 [52.0;90.2]	0.653	192
**Lowest body temperature** (deg C)	33.2 [32.1;33.8]	33.4 [32.1;33.9]	33.0 [32.1;33.7]	0.260	192
**Operation duration** (min)	234 [195;276]	238 [200;274]	227 [181;276]	0.147	192

**Table 2:** Pre- and postoperative levels and changes in median of troponin and total ketone bodies. *p*-value shows stratification after procedures with and without cardiotomy. Median and interquartile range (IQR) are shown as summary measures. For inference regarding perioperative changes, the change in median and the associated 95% confidence intervals shown are based on the paired-sample Wilcoxon test.

	All Patients	No Cardiotomy	With Cardiotomy	*p*
	*n* = 192	*n* = 59	*n* = 133	
Preoperative (descriptive) [Median; IQR]				
Troponin (ng/L)	14.0 [10.0; 23.0]	14.5 [10.0; 23.0]	14.0 [10.0; 24.0]	0.962
Total Ketone Bodies (µM)	302 [144; 552]	267 [137; 526]	337 [148; 577]	0.378
Postoperative (descriptive) [Median; IQR]				
Troponin (ng/L)	317 [219; 534]	236 [170; 349]	358 [242; 640]	<0.001
Total Ketone Bodies (µM)	154 [85; 362]	173 [102; 390]	154 [79; 336]	0.398
Change (postoperative–preoperative, descriptive) [Median; IQR]				
Troponin (ng/L)	286 [204; 511]	216 [135; 310]	329 [228; 624]	<0.001
Total Ketone Bodies (µM)	−87 [−311; 20]	−78 [−285; 28]	−98 [−348; 20]	0.734
Change (postoperative–preoperative, inference) [Change in median; 95% confidence interval]				
Troponin (ng/L)	340 (302 to 391, *p* < 0.001)	233 (200 to 274, *p* < 0.001)	411 (350 to 496, *p* < 0.001)	
Total Ketone Bodies (µM)	−123 (−175 to −79, *p* < 0.001)	−119 (−223 to −37, *p* = 0.003)	−126 (−191 to −77, *p* < 0.001)	

## Data Availability

Not available.

## Supplementary Materials

The following supporting information can be downloaded at: https://www.mdpi.com/article/10.3390/cells13040294/s1, Figure S1: Linear relationship between the change in total keton bodies and log-transformed change in troponin levels stratified according to cardiotomy; Figure S2: As in Figure 4 of the main manuscript, but stratified according to cardiotomy; Table S1: Regression coefficients of a linear regression with the log-transformed change in troponin levels as outcome; Table S2: Regression coefficients of a linear regression with the change in total ketone bodies and the log-transformed change in Troponin levels.
