# Supplementary material for: Levels of Circulating Ketone Bodies in Patients Undergoing Cardiac Surgery on Cardiopulmonary Bypass
Source: Cells. 2024 Feb 6;13(4):294. doi: 10.3390/cells13040294 (PMC10886663; doi:10.3390/cells13040294)
Supplement: Supplementary file 1 [file cells-13-00294-s001.zip › cells-2805629-supplementary.pdf]

cells

Supplementary Materials

Levels of Circulating Ketone Bodies in Patients undergoing Cardiac Surgery on Cardiopulmonary Bypass

Anja Levis [1,*], Markus Huber [1], Déborah Mathis [2], Mark G. Filipovic [1], Andrea Stieger [3], Lorenz Räber [4], Frank Stueber [1] and Markus M. Luedi [1,3]

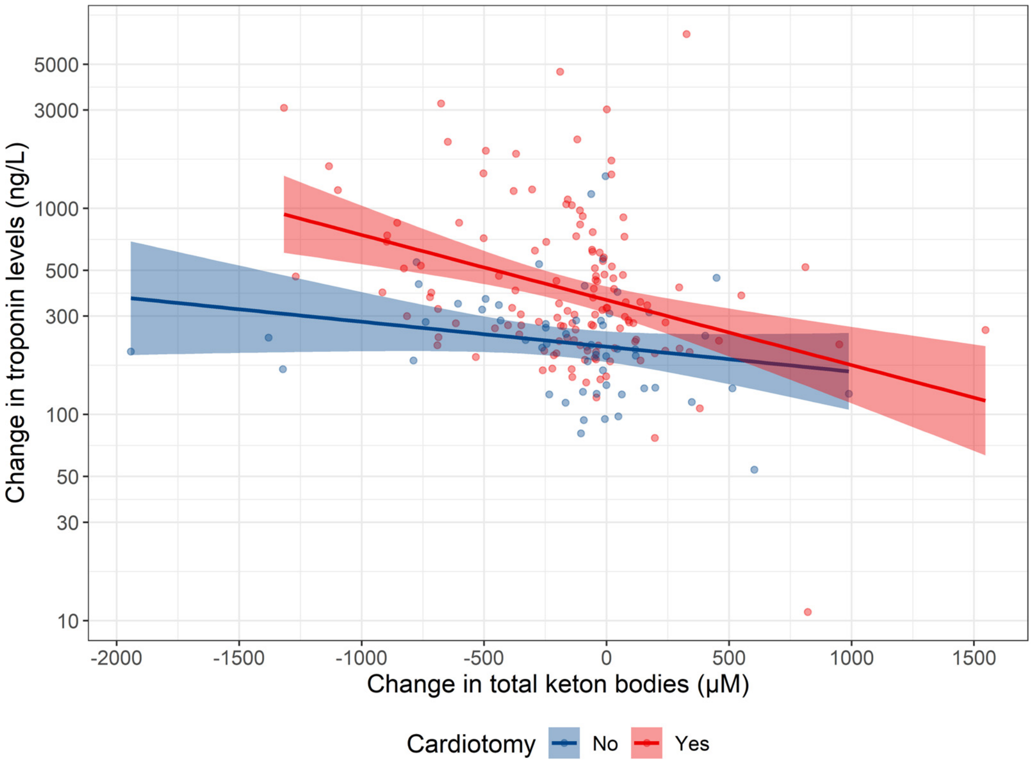

Figure S1. Linear relationship between the change in total keton bodies and log-transformed change in troponin levels stratified according to cardiotomy. The mean (solid line) and 95%-confidence interval (shaded bands) of a linear regression model are shown (the corresponding regression coefficients are shown in Supplementary Table 1).

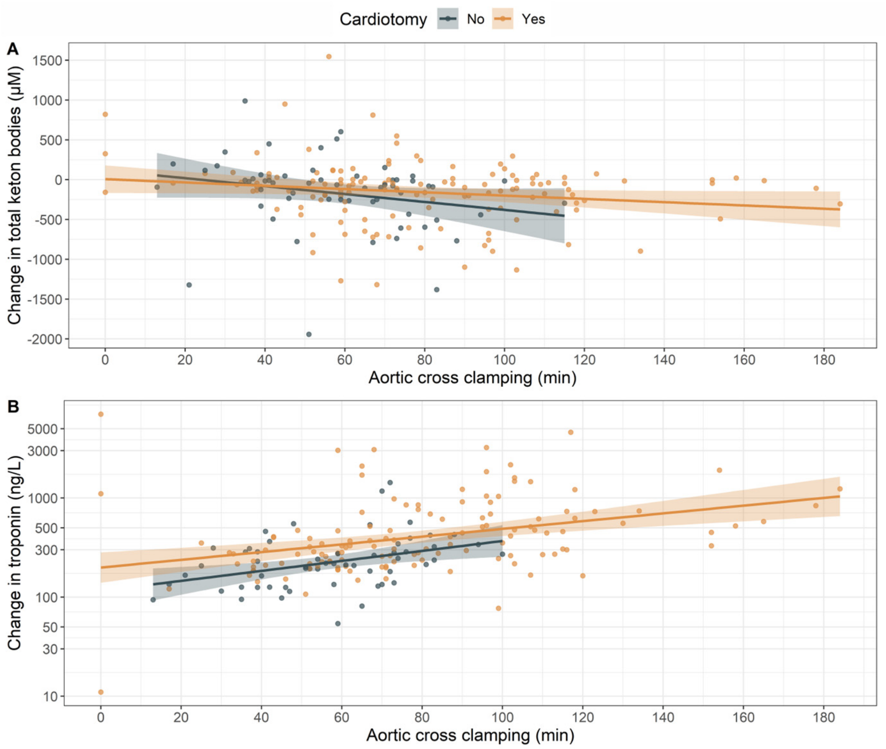

Figure S2. As in Figure 4 of the main manuscript, but stratified according to cardiotomy. The corresponding regression coefficients are shown in Table S2.

Characteristic	Beta	95% CI[1]	p-value
**Cardiotomy**			
No	—	—	
Yes	0.22	0.12, 0.34	<0.001
**Change in total keton bodies** (µM)	−0.00012	−0.00031, 0.00006	0.2
**Cardiotomy * Change in total keton bodies** (µM)			
**Cardiotomy** (Yes) * **Change in total keton bodies** (µM)	−0.00019	−0.00043, 0.00004	0.10

[1]CI = Confidence Interval

Table S1. Regression coefficients of a linear regression with the log-transformed change in troponin levels as outcome.

	Change in Total Ketone Bodies			Change in Troponin (log10)		
**Characteristic**	**Beta**	**95% CI[1]**	**p-value**	**Beta**	**95% CI[1]**	**p-value**
**Cardiotomy**						
No	—	—		—	—	
Yes	−112	−474, 250	0.5	0.23	−0.06, 0.53	0.12
**Aortic cross clamping** (min)	−5.0	−10, 0.12	0.056	0.01	0.00, 0.01	0.023
**Cardiotomy * Aortic cross clamping** (min)						
**Cardiotomy** (Yes) * **Aortic cross clamping** (min)	2.9	−2.6, 8.4	0.3	0.00	−0.01, 0.00	0.6

[1]CI = Confidence Interval

Table S2. Regression coefficients of a linear regression with the change in total ketone bodies and the log-transformed change in Troponin levels.
